# Development and evaluation of a cancer-related fatigue patient education program: protocol of a randomized controlled trial

**DOI:** 10.1186/1472-6955-7-12

**Published:** 2008-07-23

**Authors:** Nina Stuhldreher, Karl Reif, Ulrike de Vries, Stefan Görres, Franz Petermann

**Affiliations:** 1 University of Bremen, Institute of Public Health and Nursing Research IPP, Department of Research on Interdisciplinary Ageing and Nursing, Grazer Str. 4, 28359 Bremen, Germany; 2 University of Bremen, Centre for Clinical Psychology and Rehabilitation, Grazer Str. 6, 28359 Bremen, Germany

## Abstract

**Background:**

Cancer-related fatigue (CRF) and its impact on patients' quality of life has been an increasing subject of research. However, in Germany there is a lack of evidence-based interventions consistent with the multidimensional character of fatigue. The objective of this study is to develop and evaluate a self-management program for disease-free cancer patients to cope with CRF.

**Methods:**

Based on evidence extracted from a literature review, a curriculum for the self-management program was elaborated. The curriculum was reviewed and validated by an interdisciplinary expert group and the training-modules will be pretested with a small number of participants and discussed in terms of feasibility and acceptance.

To determine the efficacy of the program a randomised controlled trial will be carried out: 300 patients will be recruited from oncological practices in Bremen, Germany, and will be allocated to intervention or control group. The intervention group participates in the program, whereas the control group receives standard care and the opportunity to take part in the program after the end of the follow-up (waiting control group). Primary outcome measure is the level of fatigue, secondary outcome measures are quality of life, depression, anxiety, self-efficacy and physical activity. Data will be collected before randomisation, after intervention, and after a follow-up of 6 months.

**Discussion:**

Because there are no comparable self-management programs for cancer survivors with fatigue, the development of the curriculum has been complex; therefore, the critical appraisal by the experts was an important step to validate the program and their contributions have been integrated into the curriculum. The experts appreciated the program as filling a gap in outpatient cancer care.

If the results of the evaluation prove to be satisfactory, the outpatient care of cancer patients can be broadened and supplemented.

**Trial Registration:**

ClinicalTrials NCT00552552

## Background

Most cancer patients experience fatigue as a symptom of their disease or as a side effect of treatment with chemotherapy, radiation therapy, immunotherapy or surgery. It is described as a subjective feeling of extreme tiredness and decreased functional status, which are not adequate to activities performed and are not relieved by sleep or rest [1]. The specific manifestations may be physical, mental or emotional [2].

A small number of patients recover from cancer-related fatigue after cessation of treatment, but in a significant proportion of cancer survivors the fatigue persists for several months or even years. Estimated rates for the prevalence of fatigue in disease-free cancer patients range from 17% to 56% subject to type and stage of cancer and the fatigue assessment instrument [3-7]. But often CRF is not diagnosed because cancer survivors rarely report it and doctors and nurses do not automatically focus on this symptom [1,8].

CRF is described as a more distressing symptom than nausea, vomiting or pain and it impairs the quality of life [8-12]. According to this, everyday life is also severely restricted: it leads to prolonged disability with consequences for the resumption of social life as well as work [13-15].

The aetiology of CRF after termination of treatment is currently the object of research. The majority of work to date suggests that the level of fatigue in disease-free cancer patients is correlated more strongly with psychosocial factors like activity level, sleep problems and psychological distress than to type of cancer or type and extent of cancer treatment [7,16-18].

The common regimen of CRF focuses on single interventions such as treatment of anaemia [15,19,20] – which actually is necessary if haemoglobin levels are low – or on increasing physical activity [21-25]. In many cases, though, this is not sufficient because the multidimensional character of CRF, and especially the psychosocial components are not addressed adequately. There is evidence that behavioural changes combined with an increase of the level of physical activity and the restructuring of daily routine as well as personal consultation result in a sustainable reduction of fatigue [1,3,12,13,26-31].

Our study, therefore, has aims on different levels: to create and test a self-management program which empowers disease-free cancer patients to cope with fatigue and to achieve an improvement in the outpatient care of cancer. In addition, the interaction between depression, anxiety and fatigue will be investigated.

Our predictions are:

1. Participation in the self-management program FIBS (Fatigue individuell bewältigen – ein Selbstmanagementprogramm [Coping with fatigue individually – a self-management-program]) significantly decreases the level of fatigue in disease-free cancer patients.

2. Participation in the self-management program FIBS increases the quality of life in disease-free cancer patients.

3. FIBS has a positive impact on participants' levels of anxiety and depression.

## Methods/design

### Development of the self-management program

To define the contents of the intervention an intensive literature review was conducted. The following were identified as essential subjects for the curriculum:

• medical background and causes of CRF

• physical activity and moderate exercise

• restructuring daily schedules

• energy conservation

• stress-management and relaxation strategies

• coping with negative emotions

• integrating the new knowledge into everyday life.

The program aims at impacting on health-related self-efficacy by the training of problem solving, goal setting, and cognitive techniques as knowledge transfer hasn't proved sufficient to achieve changes in behaviour. According to this we developed a curriculum in which detailed information is provided for every module concerning objectives, background, methods and materials. The program will be administered by qualified nurses to groups of eight participants. It includes six weekly sessions à 90 minutes each dealing with one of the topics listed above.

### Formative evaluation

The curriculum was reviewed and validated by an expert group of oncologists, psycho-oncologists, nurses, social workers, physical therapists, health scientists, and patients. The tutors were trained with these results. The preliminary modules were tested with a small number of patients and discussed regarding feasibility, acceptance and improvements. After revision by the expert group and the research team, the program was implemented.

### Summative evaluation

#### Design and setting

**Figure 1:**
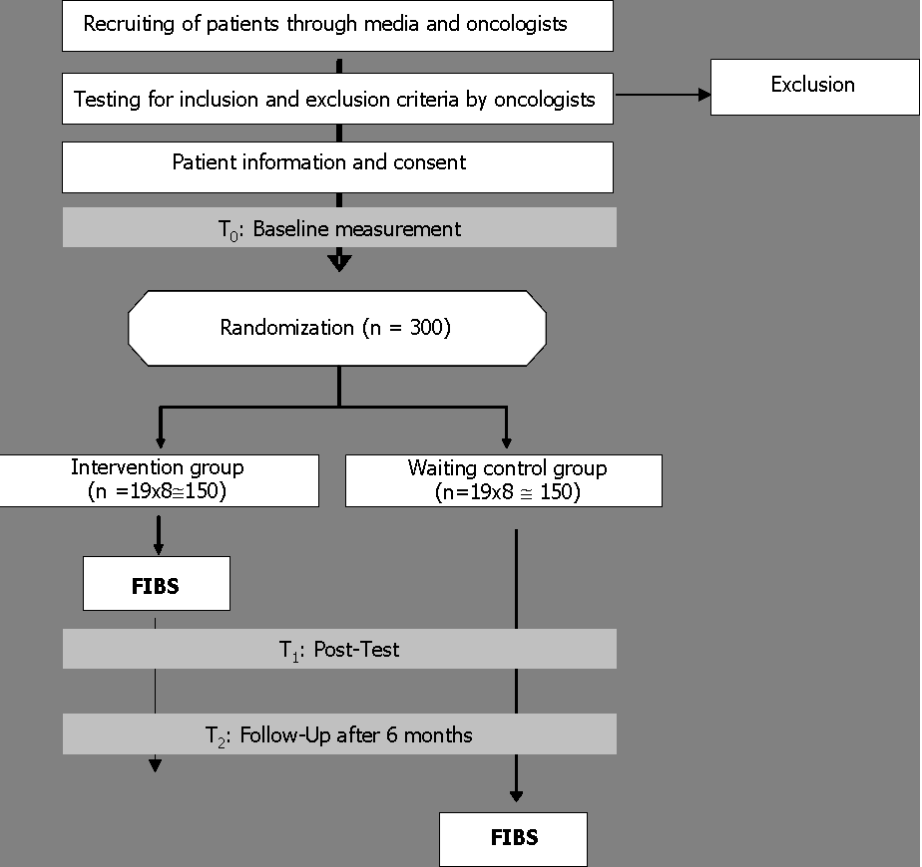
Summary of trial design.

A randomized controlled trial with intervention and a control condition will be applied to evaluate the efficacy of our self-management program to reduce the level of fatigue in disease-free cancer patients. The study is conducted at the information centres for cancer in Bremen, Germany. A summary of trial design is given in figure 1.

#### Identification of eligible patients/Inclusion and exclusion criteria

The recruitment of the patients will be carried out by oncologists in Bremen. The practices are informed about our study through participation in the expert group as well as through publications in relevant medical journals and personal information from a member of the research team. Criteria for inclusion in the study are: Patients must be over18 years of age and diagnosed with malignant tumours. They should have finished active treatment be in a stable condition with an ECOG performance status [32] of 0–2. Their level of fatigue should be rated as moderate (4–6) or severe (7–10) on a scale from 0–10. Formally, exclusion criteria are a life expectancy less than 12 months, profound brain tumours or metastases, cognitive disorders or severe psychiatric conditions.

Eligible patients will be informed of the ongoing study and asked by their oncologists to participate. They will be invited to a briefing in which they will be informed in detail about objectives and study procedures. Subsequently they will be asked to give their written informed consent.

#### Randomization

Participants are continually randomized to the self-management program or a waiting control group. Members of the waiting group receive the intervention after the intervention group has completed their follow-ups, which will be about six months after inclusion in the study. The allocation to each group will be based on a random list with balanced bloc randomization. The randomization will take place each time 16 study participants are eligible. In terms of concealment, patients will be informed of their assignment by phone.

#### Blinding

As consequence of the design with intervention compared to no intervention blinding of the participants is futile. But to avoid any bias, data entry and analysis will be performed by neutral researchers who are blinded to group allocation.

#### Data Collection and Outcome measures

Data will be collected three times by self-rated measures: Baseline data will be collected before patients are randomized to one group. The second measurement will take place each time after an intervention has taken place for the intervention and its associated control group. A follow-up will be carried out six months later.

Fatigue is the primary outcome measured by the Fatigue Assessment Questionnaire (FAQ) [33]. The instrument differentiates between physical, cognitive and affective fatigue and defines the level of fatigue on a scale from 0 to 60.

Secondary outcomes to test the behavioral impact of the intervention are general self-efficacy [34], self-efficacy of physical activity [35], levels of physical activity [36], and knowledge about fatigue and its therapy.

Further outcomes which are potentially related to/affected by fatigue are anxiety and depression measured by the German version of the hospital anxiety and depression scale (HADS) [38] and quality of life by the EORTC QLQ-C30 [39].

To control for differences between the groups and possible confounders, several sociodemographic variables and medical background information are also recorded.

Outcomes are measured at three different points in time: before randomization; after completion of the program, which is about six weeks later; and after a follow-up at six months.

#### Sample size considerations

In terms of detecting a difference in FAQ scorings of 4 points with a median of 15 (alpha = 0.05; beta = 0.2) and 25% estimated rate of drop-outs, 300 patients (150 in each group) have to be recruited; therefore, about 19 randomization processes, as described above, have to be carried out over the entire term in order to assemble groups of 8 participants.

The effect size d was calculated as a result of inconsistent presumptions regarding the other outcomes describing the expected effects and standard deviations. If 300 patients are included, a small to moderate effect size of 0.35 can be detected in a two-tailed test setting alpha = 0.05 and beta = 0.2, which is assumed to be satisfactory.

#### Data analysis

Data analysis will be carried out according to an analysis protocol following the "intention to treat principle." The primary analysis will be a comparison of the levels of fatigue in the intervention and the control groups and their changes over the course of time. Logistic regression models and variance analyses will be used to determine the impact/effect modification of secondary outcomes and to control for possible differences between the groups. All significance tests will be two-tailed and chosen allowing for scale levels.

### Ethical considerations

Full ethical approval for this study has been obtained by the ethical committee of the University of Bremen, Germany.

### Time plan

Development and formative evaluation of the intervention began in April 2007 and was finished in January 2008. Patient recruitment therefore began in February and will continue until February 2010. The study will be completed in June 2010.

## Discussion

FIBS is expected to be an effective intervention to control fatigue by achieving behavioural changes in disease-free cancer patients.

## Abbreviations

CRF: Cancer-related Fatigue; FIBS: Fatigue individuell bewältigen – ein Selbstmanagementprogramm (Coping with fatigue individually – a self-management-program); FAQ: Fatigue Assessment Questionnaire; HADS: Hospital anxiety and depression scale.

## Competing interests

The authors declare that they have no competing interests.

## Authors' contributions

KR, SG and FP were responsible for defining the research question and the drafting of the study proposal. KR, UdV and NS are integrated into coordination and realization of the study. NS and KR were responsible for the drafting of this paper, although all authors read and approved the final version.

## Pre-publication history

The pre-publication history for this paper can be accessed here:


